# Accessory Maxillary Sinus Ostium Frequency and Correlation with Anatomical Variables and Sinus Mucosal Status: A CBCT Study 

**DOI:** 10.30476/dentjods.2025.104073.2502

**Published:** 2025-12-01

**Authors:** Seyyed Hosein Rudbarizade, Fereshteh Goudarzi, Kianoosh Malek Zadeh, Masoomeh Afsa

**Affiliations:** 1 Dentist, Bandar Abbas, Iran.; 2 Dept. of Oral and Maxillofacial Radiology, Faculty of Dentistry, Hormozgan University of Medical Sciences, Bandar Abbas, Iran.; 3 Dept. of Medical Genetics, Faculty of Medicine, Hormozgan University of Medical Sciences, Bandar Abbas, Iran.

**Keywords:** Cone-Beam Computed Tomography, Maxillary sinus, Maxillary Ostium

## Abstract

**Background:**

The accessory maxillary sinus ostium (AMO) is one of the anatomical variations in the maxillary sinus. The acquired or congenital nature of AMO has not been proven. In theory, mucus drained from the principal ostium may reenter the sinus through the accessory ostium and cause maxillary rhinosinusitis.

**Purpose:**

The aim of the present study is to investigate the AMO frequency and its correlation with some anatomical and pathological variables in the area using cone-beam computed tomography (CBCT) images.

**Materials and Method:**

This was a descriptive-analytical cross-sectional study. CBCT images were obtained from 273 individuals and a total of 461 maxillary sinuses. The presence of AMOs and their relationship with age, sex, sinus mucosa condition, patency of the principal ostium, septal deviation, and sinus dimensions were measured.

**Results:**

The AMO frequency was 35.6% and 14.63% of sinuses had more than one AMO. AMO was more common in men. There was a statistically significant relationship between AMO presence and abnormal mucosal status of maxillary sinus. The present study showed a statistically significant relationship between the presence of AMO and the anterior-posterior dimension of the sinus.

**Conclusion:**

AMO occurred more frequently in the sinuses with abnormal mucosal status. However, when the abnormal sinus mucosa has reached the nasal fontanelle, it is not possible to check the presence of AMO by CBCT images.

## Introduction

Paranasal sinuses begin to develop as depressions from the nasal cavity into the corresponding bones (maxilla, frontal, sphenoid, and ethmoid) during the embryonic period and continue to grow until skeletal maturity. Like the nasal cavity, the paranasal sinuses are covered by respiratory epithelium, which is composed of pseudostratified ciliated columnar cells. These cilia push the sinus secretions into the nasal cavity through the principal maxillary ostium (PMO) and the ostiomeatal complex (OMC) [ 1 ].

The medial wall of the maxillary sinus, which houses the OMC, consists of the maxilla, ethmoid, inferior turbinate and palatine bones [ 2 ]. PMO is located at the junction of the medial wall of the maxillary sinus and the floor of the orbit, and the sinus secretions are discharged through this ostium first to the hiatus semilunaris, then to the middle meatus, and finally to the nasal cavity. Therefore, the patency of this ostium and its drainage path play an important role in maintaining the health and physiological function of the sinus [ 3 - 4 ].

Accessory maxillary ostium (AMO) is any opening other than the principal ostium, which is usually located in the nasal fontanelle or hiatus semilunaris. The nasal fontanelle is located in the middle meatus below the uncinate process and above the inferior turbinate and consists of the nasal mucosal membrane, maxillary sinus mucosal membrane, and a layer of connective tissue [ 5 ]. Sinus secretions are transferred only through PMO, and AMO plays no role in the physiological drainage of mucus. But this drained mucus may enter the sinus again through the AMO (mucus recirculation). For this reason, one hypothesis suggests that the presence of AMO may play a role in the development of chronic maxillary rhinosinusitis [ 6 ].

It is not clear whether AMO is acquired or congenital. When the function of sinus-ciliated cells is impaired, sinus drainage is disturbed, and mucus accumulation causes a subsequent sinus infection. Infection increases mucus accumulation and, finally, leads to PMO obstruction. One hypothesis shows that following increased mucus accumulation and subsequent infection and failed secretion drainage, elevated sinus pressure occurs, which eventually leads to the rapture of the weak membrane of the medial sinus wall and eventually the formation of AMO. According to this hypothesis, AMO formation is similar to the perforation of the ear tympanic membrane due to an acute middle ear infection [ 6-8 ]. Nitric oxide produced by sinus epithelium cells has antibacterial properties [ 9 ]. Theoretically, since the AMO formation increases the sinus ventilation rate and the return of the sinus drainage through the principal ostium into the sinus, the concentration of the resulting nitric oxide is reduced, which can probably cause pathological changes such as increasing the mucus thickness, the formation of mucous retention cysts, and sinusitis [ 6,9].

The results of Soylemez *et al*. [ 10 ] demonstrated that AMO was very rare in patients younger than 13 years old (2 out of 142 patients) compared with 100 out of 142 patients older than 13 years old. This finding indicates that AMO is more likely to form after completion of sinus development.

Since there are questions about the origin and role of the accessory ostium and its relationship with sex, age, and changes in the sinus mucosa and the results of previous studies are contradictory, the present study aimed to investigate the presence of AMO using cone beam computed tomography (CBCT) images as well as its relationship with possibly influential factors such as age, sex, sinus mucosa changes, principal ostium patency, sinus dimensions, and nasal septal deviation.

## Materials and Method

**Figure 1:**
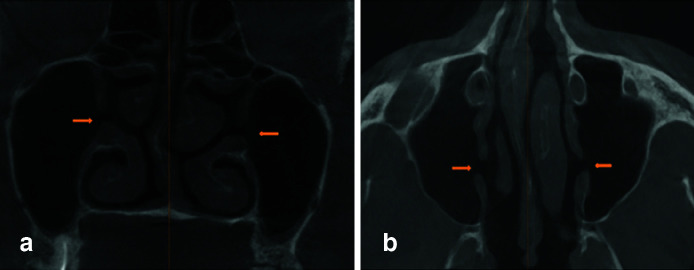
Accessory maxillary sinus ostium (AMO) in **a:** Coronal section and, **b:** Axial section

CBCT images of patients who had been referred to a private maxillofacial radiology center for various diagnostic purposes between January 2020 and September 2022 were examined in the present retrospective study. The inclusion criteria were age over 18 years, complete image of the maxillary sinuses and nasal cavity in the CBCT field of view, and the appropriate quality of the images in the sinus area. Exclusion criteria also included the history of trauma, pathology, and surgery in the sinuses and midface. CBCT images were prepared by the Pax-Duo 3D Vatech machine. The exposure conditions, including KVp and mA, were selected taking into account the patient's size based on the device settings. Then, CBCT images in the axial and coronal planes were examined by a maxillofacial radiologist using EZ3D viewer software. The presence and number of accessory ostium, and opened or obstructed principal ostium were recorded in each sinus (Figure 1). The cases that met the inclusion criteria but whose determination of the presence of accessory ostium was not possible due to the increased density of the sinus space in the vicinity of the nasal fontanelle were also recorded as unknown cases. To determine the mucosal morphology of the sinus, the classification of Soikkonen and Ainamo's [ 11 ] was employed, which is defined as (1) invisible mucus membrane or with sinus mucosal thickness (SMT) less than 2mm, (2) SMT greater than 2mm with a smooth pattern, (3) SMT greater than 2mm with a dome pattern (suspected mucous retention cyst), (4) SMT greater than 2mm with a folded pattern, and (5) completely opacified sinus.

Nasal septa were examined in coronal and axial sections and were divided into non-deviated straight septum, right, left, S-shaped, and reverse S-shaped septal deviations.

Anteroposterior (AP), mediolateral (ML), and superoinferior (SI) dimensions were recorded in millimeters using the linear measurement tool of EZ3D Plus software. Sinus volume was also calculated based on the formula presented in Sharma *et al*.'s study [ 12 ] by multiplying AP, ML, and SI dimensions by 0.52. 

All evaluations were done by a maxillofacial radiologist with nearly 15 years clinical examination. In case of uncertainty, another maxillofacial radiologist was consulted to reach a consensus.

Data analysis was carried out using SPSS version 25 (IBM Corporation, Armonk, NY, USA). Qualitative data were reported as frequency and percentage, and quantitative data were reported as mean and standard deviation. The chi-square test and ANOVA were used to measure the relationship between variables. p value< 0.05 was considered statistically significant. 

## Results

A total of 148 (62.44%) studied subjects were women, and 89 (37.55%) were men. The mean age was 32.96 years, and the age range was 18–79 years. A total of 237 CBCT images, including 461 maxillary sinuses, were evaluated. In seven scans, only the right sinuses were assessed; in six scans, only the left sinuses were examined; and in 224 scans, both the right and left sinuses were evaluated.

AMO was found in 164 sinuses (35.6%), of which 85 cases (18.43%) were related to the right sinus and 79 cases (17.13%) were related to the left sinus. A total of 140 sinuses (30.36%) had one AMO, 21 sinuses (4.6%) had two AMOs, and 3 sinuses (0.7%) had three AMOs. 

**Figure 2:**
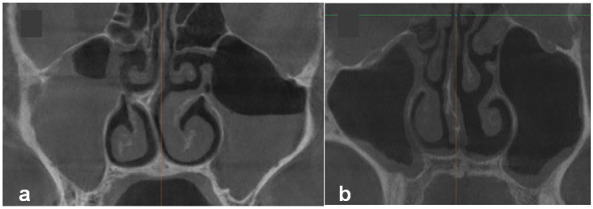
In the case of sinus opacification or **a:** Peripheral sinus mucosal thickening, **b:** The presence of accessory maxillary sinus ostium (AMO) is not identifiable

Also, the presence or absence of AMO could not be determined in 19 cases (14.63%) of all sinuses due to complete sinus opacification or an increase in the SMT peripherally (Table 1) (Figure 2).

**Table 1:** Demographic and accessory maxillary sinus ostium (AMO) distribution

			Count
Sex	Female	292	461
	Male	169	
Sinus Side	Right	231	461
	Left	230	
AMO	With AMO	164	461
	Without AMO	278	
	Non Identifiable	19	
Age	Min.	18	
	Max.	79	
	Mean	32.96	

Intraobserver agreement for AMO presence, PMO patency, and septal deviation were 0.84, 0.82 and 0.92, respectively that were strong based on Kappa statistics classification.

### AMO and age, sex, and side

**Figure 3:**
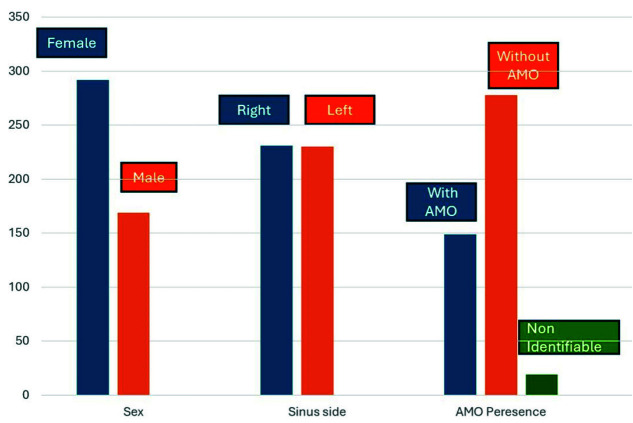
Demography and accessory maxillary sinus ostium (AMO) distrubution

Age and sinus side were not influencing factors in the presence of AMO (*p*= 0.361 and *p*= 0.075, respectively). However, sex seemed to be an influencing factor in the presence and absence of AMO (Table 2) (Figure 3).

**Table 2:** Distribution of sinuses with accessory maxillary sinus ostium (AMO) in males and females

	With AMO	Without AMO	Non Identifiable	Count	*p* Value
Female	95	188	9	292	0.42
Male	69	90	10	169	

### AMO and nasal septal deviation

A straight nasal septum was observed in 31 patients (n= 62 sinuses, 13.44%), among whom AMO was present in 8 right sinuses and 8 left sinuses (3.4%).

The right septal deviation was observed in 193 sinuses. In this group, AMO was present in 43 right sinuses (9.32%) and 38 left sinuses (8.24%). Left septal deviation was also observed in 193 sinuses, where AMO was found in 32 right sinuses (6.94%) and 30 left sinuses (6.5%).

The right septal deviation was observed in 99 right sinuses (21.47%) and 94 left sinuses (20.39%). In this group, AMO was present in 43 right sinuses (9.32%) and 38 left sinuses (8.24%).

Left septal deviation was also observed in 95 right sinuses (20.6%) and 98 left sinuses (21.25%), where AMO was found in 32 right sinuses (6.94%) and 30 left sinuses (6.5%).

S-shaped septal deviation was observed in three patients (six sinuses), where AMO was found in one right sinus and two left sinuses. Moreover, reverse S-shaped septal deviation was observed in three right sinuses and four left sinuses, where AMO was present in one right sinus and one left sinus. In general, there was no statistically significant difference between different septal deviations in terms of the presence of AMO in the right and left sinuses (*p*= 0.59 and *p*= 0.47 in the right and left sinuses, respectively).

### AMO and Patency of PMO

Of the 461 examined sinuses, 36 sinuses (7.8%) had obstructed PMO, among which AMO was present in three sinuses (0.65%) and absent in 16 sinuses (3.47%). It was also not possible to determine the presence of AMO in 17 sinuses (3.68%) due to the complete opacification or the peripheral increase in SMT. PMOs were open in 425 (92.19%) sinuses, of which 161 sinuses (34.92%) had AMO and 262 sinuses (56.83%) did not have AMO. It was also not possible to determine the presence of AMO with certainty in two cases due to sinus opacification and the peripheral increase in SMT. Sinuses with patent PMO and obstructed PMO were significantly different in terms of AMO presence and absence (*p*< .001).

### AMO and SMT

**Figure 4:**
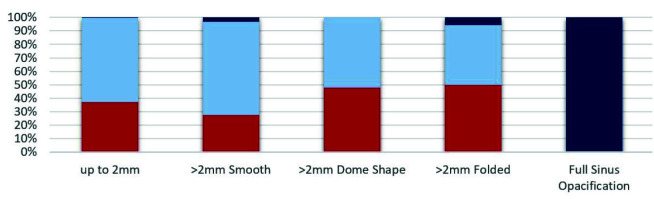
Distribution of Sinuses with accessory maxillary sinus ostium (AMO) based on sinus mucosal thickness

Out of 461 examined sinuses, SMT was more than 2mm in 171 sinuses (31.09%). Among these, AMO was present in 56 sinuses (12.14%) and absent in 97 sinuses (21.04%). It was also not possible to determine the AMO presence in 18 sinuses (3.9%). Out of 290 (62.9%) sinuses with normal SMT (invisible SMT up to 2mm SMT), 108 sinuses (23.42%) had AMO, and 181 sinuses did not have AMO. It was also not possible to determine the presence of AMO in one sinus. In sinuses with normal mucosal status (SMT less than 2mm), the number of sinuses with AMO was significantly higher than sinuses with abnormal mucosal status (*p*< 0.001) (Table 3) (Figure 4).

**Table 3:** Distribution of sinuses with accessory maxillary sinus ostium (AMO) based on sinus mucosal status

	With AMO	Without AMO	Non Identifiable	Count	*p* Value
up to 2mm	108	181	1	290	0.000
>2mm Smooth	26	66	3	95	0.122
>2mm Dome Shape	21	23	0	44	0.103
>2mm Folded	9	8	1	18	0.373
Full Sinus Opacification	0	0	14	14	0.000

### AMO and Measurements of Maxillary Sinus

A statistically significant relationship was found between the AP dimension of the sinus and the presence of AMO (*p*= 0.03). That is, as the AP dimension increases, the possibility of AMO presence increases. There was no significant relationship between SI, ML, and sinus volumes with AMO presence.

## Discussion

In the present study, AMO was found in 35.6% of the sinuses, 85.36% of which had one AMO, and 14.63% of the sinuses had more than one to a maximum of three AMOs. The AMO frequency in different studies has been reported to range from 24.7% to 47.2% [ 13 - 20 ]. Also, the number of sinuses with more than one AMO was reported from 12.2% to 39.5% [ 13 - 14 ]. Consistent with the present study, the highest AMO number in one sinus was also reported to be three in other studies [ 12 , 14 ]. In the present study, the AMO frequency was higher in women. Likewise, Yeng *et al*. [ 14 ] have reported that the AMO frequency was higher in women. However, Kuofeng Hung [ 13 ], Majid Bani Ata [ 16 ], Ozel [ 19 ] and Shishir Shetty [ 20 ] *et al*. reported no statistically significant relationship between AMO presence and sex. This difference in our study can be attributed to the higher number of females compared to males (292 females vs. 169 males).

The present study, as well as previous studies, showed no relationship between AMO presence and age [ 13 - 14 , 19 - 20 ]. However, due to the lack of available CBCT for people under 18 years of age in our archive, this age group was not included in the present study. The results of the study by Soylemz *et al*. [ 10 ] showed that the occurrence of AMO is rare in patients aged <13 years, suggesting that there may be a nasal fontanelle perforation in patients with sinusitis or principal ostium obstruction.

The results of the present study showed no statistically significant relationship between the presence or absence of septal deviation as well as the deviation direction and the AMO presence in the right or left sinuses. Contrary to this, Ozel *et al*. [ 19 ] showed a higher probability of AMO in people with septal deviation, as well as the highest AMO number in the sinuses that had the same direction as the septal deviation. This discrepancy may be due to the significant difference in the sample size, which were 807 people in the above study and 237 people in the present study.

In the present study, PMO obstruction occurred in subjects with localized mucosal status in the infundibulum, complete opacification of the maxillary sinus, or partial opacification of the maxillary sinus up to the ostiomeatal complex level in such a way that it caused infundibulum obstruction or an increase in SMT peripherally (Figure 2). In total, 36 sinuses had obstructed PMO, three of which had AMO. AMO was not observed in 16 cases, and it was not also possible to investigate the AMO presence due to an increase in SMT or the observation of sinus opacification in the vicinity of the nasal fontanelle. These findings are contrary to the results of the study by Soylemz *et al*. [ 10 ], which reported a significant relationship between the PMO obstruction and AMO presence. One of the features of sinusitis in computed tomography (CT) and CBCT imaging is sinus opacification due to the accumulation of secretions and the increase in SMT resulting in principal ostium obstruction as the sinus drainage path [ 10 , 15 - 16 , 21 - 24 ]. However, when these conditions are observed in CBCT, the nasal fontanelle cannot be studied for the presence of AMO, which indirectly contradicts the results of studies that have reported a direct relationship between sinusitis and the AMO presence.

In the present study, in 171 sinuses with SMT> 2mm, 56 sinuses (32/7%) had AMO and 56/7% did not have AMO. In 290 sinuses with SMT<2mm, 37/2% had AMO and 62/4% lacked AMO. The Chi-square test revealed a statistically significant relationship between SMT and AMO presence. However, it may contribute to the difference in the number of sinuses with non-identifiable AMO due to sinus opacification around the fontanelle (Table 3).

The results of the present study did not show any statistically significant difference between the AMO presence in different patterns of morphological changes in the maxillary sinus mucosa, which was similar to the results of the study by Kuo Feng Hung *et al*. [ 13 ] and Soylemz *et al*. [ 10 ].

With the assumption that the bigger dimensions of the maxillary sinus may be an effective factor in the occurrence of AMO, the dimensions were also investigated, and the results showed a statistically significant relationship between the AP dimension and the AMO presence. In other words, the AMO frequency was higher with an increase in the AP dimension. Meanwhile, there was no statistically significant relationship between any of the SI, ML dimensions, and sinus volume with the AMO presence. It is not yet definitely known whether AMO is congenital or acquired; hence, investigating this relationship in future studies with larger sample sizes would be helpful.

There are some conditions like smoking, exposure to environmental factors, allergy and so on that increase the risk of sinusitis. It is suggested to design studies to compare these factors in cases and control groups to see if these factors can also have multiplying effect on AMO formation. The other point is the unknown effect of AMO on the treatment of sinusitis that can be evaluated in future studies.

## Conclusion

In this retrospective study, the AMO frequency was 35.6%, of which 14.63% had more than one to three A-MO numbers. AMO was observed only in a small number of blocked PMO cases. The AMO frequency was higher in women. There was a correlation between the AMO presence and abnormal mucosal status. The present study showed a relationship between the AMO presence and the anterior-posterior dimension of the sinus.
